# Workplace Violence Against Nurses in Canada: A Legal Analysis

**DOI:** 10.1177/15271544231182583

**Published:** 2023-07-04

**Authors:** Sioban Nelson, Kathleen Leslie, Aleah McCormick, JohnPaul Gonsalves, Andrea Baumann, Natalie J. Thiessen, Catharine Schiller

**Affiliations:** 1 Bloomberg Faculty of Nursing, 7938 University of Toronto, Toronto, ON Canada; 2 Faculty of Health Disciplines, 1349 Athabasca University, Athabasca, AB Canada; 3 Faculty of Law, University of Toronto, Toronto, ON, Canada; 4 School of Nursing, University of Northern British Columbia, Prince George, BC Canada

**Keywords:** workplace violence, occupational health, criminal law, nursing, delivery of health care, liability, employment, government, Canada

## Abstract

Workplace violence against nurses is a significant global occupational health problem, with incidents of violence increasing in frequency since the COVID-19 pandemic began. In this article, we provide a review of recent legislative amendments meant to bolster workplace safety in health care in Canada, analyze legal cases where nurses were the victims of violence, and discuss what these legal reforms and decisions reveal about how nurses’ work is treated within the Canadian legal system. Under criminal law, the limited number of cases we could find with oral or written sentencing decisions show that, historically, the fact a victim was a nurse was not always considered an aggravating factor on sentencing. Recent legislative amendments make this a specified aggravating factor and it is important to track the impact of these amendments when judges exercise their discretion in sentencing. Under employment law, it appears that, despite the government's efforts to increase the deterrence factor under legislation with significantly increased fines for employers who fail to protect their employees from injury, courts remain reluctant to impose such sanctions. In these cases, it is also important to track the impact of harsher penalties. We conclude that combating the widespread normalization of workplace violence in health care, and specifically against nurses, is acutely needed to help ensure that these ongoing legal reforms aimed at improving the safety of health workers are effective.

## Introduction

Workplace violence against nurses is a significant global occupational health problem widely acknowledged in the academic and policy literature (International Council of Nurses [ICN], 2017; Li et al., 2020; Lu et al., 2020; Spector et al., 2014). Nurses experience intimidation, threats, and violence far too often in the course of their employment. A recent survey by four international health organizations found that violence against health care workers has been occurring at a higher frequency since the COVID-19 pandemic began in 2020, and that it happens all over the world (ICN et al., 2022).

Workplace violence is a major concern for nurses in Canada and has received increasing attention from Canadian nursing organizations and government stakeholders (British Columbia Nurses Union [BCNU], 2019; Canadian Federation of Nurses Unions [CFNU], 2017; CNA, 2019; Canadian Nurses Association [CNA] & CFNU, 2014; Casey, 2019; Government of Canada, 2021; Registered Nurses’ Association of Ontario [RNAO], 2019). In this paper, we review recent legislative amendments meant to bolster safety in health care environments in Canada and analyze court cases where nurses were the victims of violence.

The concept of workplace violence has been subject to much scholarly and policy discussion in recent years. Definitions of workplace violence vary widely in the academic and policy literature, making it difficult to know the full extent of the problem and to compare rates of violence across jurisdictions, and there have been calls to standardize definitions used in studying this issue (Casey, 2019; Phillips, 2016). While at one time the scope of the topic was limited to that of physical violence, discussions have broadened to include sexual violence, harassment, and other forms of psychological or emotional violence (Canadian Nurses Association & CFNU, 2014; International Labour Office, World Health Organization, International Council of Nurses and Public Services International, 2002). Scholars have also widened the discussion of workplace violence in health care to structural violence arising from power inequities acknowledging that not all health care workers are positioned equally, and some may be disproportionately affected by workplace violence (Banerjee et al., 2012; Choiniere et al., 2014).

Despite the evolution in how workplace violence is understood, its definition under occupational health and safety legislation tends to be limited to physical violence. For example, Ontario's *Occupational Health and Safety Act* (OHSA, 1990) defines workplace violence as: “the exercise of physical force by a person against a worker, in a workplace, that causes or could cause physical injury to the worker, an attempt to exercise physical force against a worker, in a workplace, that could cause physical injury to the worker, [or] a statement or behaviour that it is reasonable for a worker to interpret as a threat to exercise physical force against the worker, in a workplace, that could cause physical injury to the worker.” Workplace harassment is addressed in a separate section of OHSA, and amendments in 2016 broadened the definition to include sexual harassment. The Registered Nurses’ Association of Ontario (RNAO), the professional association representing registered nurses in Ontario, has advocated for an expanded definition of workplace violence in OHSA to include all aggressive behaviour against workers, whether physical, psychological, verbal, sexual, or emotional (RNAO, 2009). The Ontario Nurses Association (ONA), the union representing registered nurses in Ontario, has also raised concerns about the emphasis on physical violence, with the lack of acknowledgement of non-physical injuries that may result from incidences of workplace violence (ONA, 2019).

For the purposes of this paper, we were interested in the way in which the Canadian legal system handled cases involving violence against nurses. We examined the commentary of the judicial decision maker in these cases about the victim's status as a nurse and discuss what this reveals about how nurses’ work is treated within the Canadian legal system. While we did not narrow our definition of violence to physical violence, the only cases that reached the courts involved physical violence.

## Background and Literature Review

The full extent of workplace violence against nurses in Canada is difficult to determine due to pervasive underreporting, the lack of a consistent definition of workplace violence across studies and policy documents, and a lack of recent comprehensive Canadian data on this issue (Casey, 2019; CFNU, 2017; Phillips, 2016). Although now over a decade and a half old, the most recent comprehensive examination of the Canadian nursing workforce took place in 2005; this data revealed that 29% of nurses providing direct care reported having been physically assaulted at work within the past year, and 44% reported emotional abuse from a patient (Statistics Canada et al., 2006). A national survey of nurses by the CFNU (2017) found that 61% of nurses had experienced workplace violence over the previous month, and two-thirds of those had considered leaving their jobs due to the experience. A 2019 survey by the CFNU (Stelnicki et al., 2020) found that 93% of nurses reported physical assault as the most frequent type of psychologically traumatic event exposure, with 46% of surveyed nurses reporting exposure to physical assault 11 or more times.

Workplace violence against nurses can have serious consequences, both for individual nurses and for the health care system. The experience of workplace violence may result in physical injury and psychological consequences for an individual nurse and may result in short- or long-term absences from work (Gerberich et al., 2004; Phillips, 2016). The results of a 2019 CFNU national survey of nurses on occupational stress found that workplace violence can have serious impacts on nurses’ mental health, including post-traumatic stress, major depressive incidence, generalized anxiety, panic, and alcohol use disorders with the potential for suicidal ideation and attempt (Stelnicki et al., 2020). The CFNU data were collected before the COVID-19 pandemic, and there is evidence that workplace stress and violence have only intensified since then (see e.g., Grady et al., 2022; ICN et al., 2022; Tomblin-Murphy & Sampalli, 2022).

Organizational and health system consequences include injuries resulting in absences from work, decreased productivity, increased absenteeism, decreased staff morale, increased employee turnover and even the loss of nurses from the profession altogether (CFNU, 2017; Deery et al., 2011; Gates et al., 2011; Gerberich et al., 2004). Further, threats of violence and intimidation of health care workers can interfere with their ability to provide safe and effective patient care (ICN et al., 2022), saw in Canada in the context of the anti-mask, anti-vaccine, and ‘Freedom Convoy’ protests in the winter/spring of 2022. In response to these events, numerous health care organizations issued statements to their staff that included warnings against wearing scrubs so they would not be identifiable to the general public as health care providers (Katawazi, 2022; Little, 2022). In Toronto, Ontario, downtown health care facilities issued similar warnings, including a memo communicating police advice that employees should not wear identifiable clothing (Taylor, 2022). Nurses are the largest group of health care workers in Canada and typically wear scrubs in most acute care settings. These precautions, made to nurses who are predominantly women, suggested that their professional work uniform risked provoking hostility from anti-government protestors (Duong & Vogel, 2022).

There has also been increased policy-level attention paid to the problem of workplace violence in health care in recent years. A 2017 awareness campaign by the CFNU (2017) included the release of a report entitled *Enough Is Enough: Putting a Stop to Violence in the Healthcare Sector*, along with associated social media campaigns (Nelson & Baumann, 2021). At the urging of CFNU and other groups, the House of Commons Standing Committee on Health conducted hearings on the issue of workplace violence against health care workers, culminating in the release of a report with nine recommendations. Included among these recommendations was a change to Canada's *Criminal Code*, which CFNU and others had requested, a change that had also been previously proposed in a private member's bill that did not advance beyond first reading (Bill C-202, 2020). The Bill had proposed that, if the victim of an assault is a health care worker, this should be considered an aggravating circumstance when sentencing the assailant.

## Legal Framework Addressing Workplace Violence Against Nurses

The legal framework for addressing serious incidents of workplace violence against nurses consists primarily of criminal law and occupational health and safety law. Forms of workplace violence that violate Canada's *Criminal Code* (1985) include assaults, sexual assaults, and criminal harassment (stalking). Occupational health and safety legislation, such as the *Canada Labour Cod*e (1985) and Ontario's *Occupational Health and Safety Act* (OHSA) (1990*)*, sets out the obligations of employers to provide safe workplaces and includes enforcement measures governments can take against employers that fail to comply. Given provincial jurisdiction over most health facilities, Ontario's OHSA is the workplace safety legislation applicable to most nursing workplaces in Ontario, the most populous province in Canada. When there are violations of either the *Criminal Code* or occupational health and safety legislation, charges can be laid against the individual or organization and the case may proceed to judicial decision-making. Court cases involve the judicial interpretation of legislation and form a body of law known as case law. In this article, we review recent amendments to this legislation meant to address workplace violence against health care providers and examine case law that involved violent acts against nurses.

## Methods for Legal Analysis

We first reviewed recent amendments to the Canadian Criminal Code and the OHSA legislation meant to address workplace violence against health care providers. Legislative bills and acts were accessed from the Canadian Legal Information Institute (CanLII) database and Canadian government websites. Amendments were descriptively summarized, focusing on content that could impact nurses and nursing practice and any provided rationale for these changes.

Second, we reviewed case law (court cases) involving workplace violence against nurses. Case law was accessed through searches of CanLII, Westlaw, and Quicklaw legal databases. These legal databases provide access to court decisions from all Canadian courts, including judicial decision makers’ written reasons for judgment or transcripts of oral reasons for judgment. In line with our objective to understand how the Canadian legal system handled cases involving violence against nurses, we searched for Canadian criminal cases and Ontario occupational health and safety cases involving workplace violence against nurses. For feasibility, we limited our review of criminal cases to the past 15 years and our review of OHSA cases to those that had occurred since specific workplace violence provisions were added in 2010. Searches were initially conducted in May 2022. All cases were “noted up” (a legal search strategy to verify cases were still relevant and had not been overturned on appeal or overruled by subsequent cases) in October 2022. Relevant cases were identified using string searches for terms (e.g., nurse, nursing, nurs*) with type-of-case limiters, searches for cases citing specific legislative provisions, and forward and backward case citation searches. Cases were read to determine relevancy and included if the facts of the case included workplace violence against nurses. We collected the following basic information from each identified case: legal case name, year of decision, facts of the case, impact on the victimized nurse, sentencing decision (for criminal cases), charges and decision (for OHSA cases), and any discussion or commentary in the decision about the victim being a nurse. Our interdisciplinary research team included nurse lawyers, law students, and nursing academics, enriching our legal analysis while allowing for discussion of potential nursing policy and practice implications.

### Criminal Law: Recent Amendments to Canada's *Criminal Code*

Following a trial or plea bargain where an accused is found guilty of a crime, there is a sentencing process to determine the appropriate penalty. When imposing a sentence, a court will consider mitigating factors that support a lesser sentence or aggravating factors that support a harsher sentence. These factors can be based on precedent or specifically required by legislation. For example, Canada's Criminal Code includes an aggravating factor when the victim belongs to a specified public-facing, high-risk, or vulnerable job, which ultimately warrants a harsher sentence. Nurses provide a critical public service and are at high risk of workplace violence because they provide direct client/patient care. In recognition of this, and after the recommendation in the 2019 House of Commons report of the Standing Committee on Health (Canada. Parliament. House of Commons, 2019; 2021), Canada's *Criminal Code* was amended to bolster the protection of health care workers from harassment and violence (Bill C-3, 2021). These amendments came into force on January 16, 2022 and included a new requirement that courts consider an assault against a health care worker to be an aggravating factor for sentencing. A new intimidation offence was also added, making it illegal to use fear and intimidation to impede people from providing or accessing health care services in Canada (Bill C-3, 2021). Such situations include those where a health care worker faces threats or other forms of violence meant to disrupt the delivery of health care (Bill C-3, 2021). This offence does not apply to peaceful protests, such as pickets set up outside of health care institutions that might have a ‘minor impact’ on access, although the ‘minor’ qualifier is not defined (Bill C-3, 2021). On January 20, 2022, just days after the new criminal offence became legally enforceable, two individuals were charged under the new sanctions with intimidating a health care worker (DeClerq, 2022).

### Sentencing Decisions in Canadian Criminal Cases Involving Assaults Against Nurses

In Canadian case law, we could find very few reported sentencing decisions where the offender assaulted a nurse during the course of the nurse's employment. Using the search strategies described above, we identified 12 reported English-language sentencing decisions between 2006–2021 where the victim was providing nursing services (see Table 1).

**Table 1.** Canadian Criminal Cases Involving Violence Against Nurses

Case Name	Year	Facts of Case	Impact on Victimized Nurse	Sentencing Decision	Discussion of Victim being a Nurse on Sentencing
R v Gelenzoski	2006	Gelenzoski pled guilty to assault. Gelenzoski was seeking medical attention following an assault on a police officer. The offender was restrained and spit on the nurse caring for him after telling health care staff that they were positive for Hepatitis C.	The nurse reported feeling traumatized and was in fear of having contracted Hepatitis C for a year before being confirmed negative for the virus. The nurse reported that the incident interfered with their ability to do their work as a nurse.	The offender's last conviction was in 1990 and he was remorseful. The offender was sentenced to 90 days in prison for the assault on the nurse.	No, but the judge did consider the pervasiveness of violence against health care workers by placing emphasis on the sentencing objective of denunciation. “Police and health care workers are on the front lines. The message that the law will not tolerate the type of behaviour that Mr. Gelenzoski has demonstrated must be sent loudly and clearly.”
R v Knight	2009	Knight was found guilty after trial for publishing defamatory libel. Knight sent a letter to the medical ethics committee falsely accusing a nurse, his ex-wife, of allegations including drug trafficking.	The nurse reported being “angered” by the false allegations.	Knight had a long criminal record and it was not his first offense against this victim. He was sentenced to 15 months conditional sentencing to be served in the community. The court of appeal changed the sentence to be served in jail, finding Knight posed a danger to the community.	No. The judge remarked, “the innocent victim is entitled to pursue her life and her profession in peace, free from false accusations that require her to defend herself and her reputation.”
R v Stard	2016	Stard pled guilty to assault causing bodily harm. Stard was seeking health care and became uncooperative. When the triage nurse turned away, Stard repeatedly punched the nurse without warning or provocation.	The nurse suffered a concussion, torn retina, and bleeding in the eye socket. The nurse suffered PTSD and experienced difficulty with memory, balance, and focusing. The victim's personality had become unpredictable and often angry. The nurse was unable to return to work.	Stard had no criminal record and was apologetic. Stard received a conditional discharge, meaning he did not have a conviction entered against him or serve time in prison.	The victim's status as a nurse was not found to be an aggravating factor. The judge commented, “people that provide service to the public, and this would include taxi drivers, bus drivers, nurses, doctors, are in a position where they should be free from attack while doing their job.”
R v Delorey	2018	Delorey was found guilty after trial for assault with a weapon. Delorey threw a cup of urine at a nurse.	No information was provided regarding impacts on the nurse.	Delorey was already serving a life sentence in a federal prison at the time of the assault and was sentenced to 12 months in prison to be served concurrently with his ongoing sentence.	No.
R v Martin,	Court of appeal decision in 2018, varying the trial decision of 2017.	Martin pled guilty to assault. Martin was a certified involuntary patient at a psychiatric hospital and was assaulting a nursing student when a nurse intervened. Martin grabbed the nurse by the face and hair, shaking and scratching the nurse's face.	No information in judgment on injuries or other impacts on nurse or nursing student.	Martin struggled with mental illness and had a significant criminal record. She was sentenced to 45 days in jail for the assault on the nurse. The Court of Appeal varied the assault sentence to run consecutively to Martin's robbery sentence.	The victim's status as a nurse was not considered an aggravating factor. The trial judge made the general remark that “persons working in the health care system and other psychiatric patients should not be subjected to this violence.”
R v Freake	2019	Freake went to a psychiatric facility after having suicidal ideation. After leaving the hospital, he emailed threats to staff members. Despite being arrested and released on an undertaking not to go to the hospital, he returned and “caused a scene.”	Victim impact statement filed by the nurse stated that the nurse wanted to feel safe at work.	Freake was found guilty of two counts of uttering a threat and two counts of breaking an undertaking and received a conditional discharge with 12 months of probation.	No.
R v Heath	2020	Heath pled guilty to assault causing bodily harm. Heath was a hospital inpatient and struck the attending nurse with a dumbbell. The nurse fell to the floor and hit her head.	The nurse suffered broken teeth, jaw, and cheekbone. She required eight staples to the back of her head and underwent multiple surgeries, including six dental reconstructions and had her jaw wired shut for six weeks. The nurse was unable to return to work and reported ongoing physical pain, depression, and panic attacks. Two nurses witnessed the attack and were also unable to return to work after one was diagnosed with PTSD and the other with anxiety.	The offender was 62 years old and had no criminal record. The offender was sentenced to 14 months in prison.	No.
R v Paulson	2020	Paulson pled guilty to assault. While being processed at a detention centre, Paulson was intoxicated and punched a nurse in the face, knocking her to the ground.	The nurse suffered a laceration to the inside of her mouth, swollen lips, a headache, and a sore shoulder and ribs.	The offender was sentenced to 30 days in jail to be served concurrently with other sentences she received for assaults she committed against others.	No.
R v Van Horlick	2020	Van Horlick went into the nurse manager's office to request that his wife, a patient at the hospital, be moved to a different unit. He assaulted and choked the nurse manager for 11 min and then assaulted another nurse who came to assist after hearing screaming.	The nurse manager suffered extensive injuries, including an acquired brain injury, whiplash, a fractured nose, chronic neck and hand pain, and PTSD. She needed multiple surgeries and was unable to work more than six hours a week. The nurse who came to assist reported missing six months of work, living in constant fear and anxiety, and feeling unsafe at work. She suffers from tenosynovitis and her severe wrist sprain causes chronic pain.	The offender was sentenced to six months imprisonment and two years probation for each count to be served concurrently.	Yes. The trial judge considered the victims’ status as nurses to be an aggravating factor for sentencing. The judge noted that the court “must also recognize that nurses… have the right to work in a safe environment. A clear and unequivocal message must be sent…. I therefore conclude that denunciation and deterrence should be the focus of the sentence.”
R v Mustafa	2021	During a wound care community nursing visit, Mustafa attempted to kiss the nurse. When the nurse refused, Mustafa placed her in a headlock, squeezed her breast and kissed her on the neck.	The nurse was scared and anxious at work after the assault and the emotional impact was described as significant.	The offender received a suspended sentence with 18 months probation.	Yes. The trial judge considered the victim's status as a nurse to be an aggravating factor for sentencing. The appeal court upheld this as an appropriate consideration.
R v JG	2021	JG was having vitals taken in a clinical assessment room when he touched and hit the nurse's breast. JG denied the accusation and suggested the nurse was coming onto him. The trial judge found the nurse a credible and reliable witness and found JG guilty of sexual assault.	No information provided.	JG was convicted of sexual assault. He received a conditional sentence and two years probation.	No.
R v DLN	2022, appeal decision setting aside trial decision of 2019	DLN (a youth) hit, scratched, and pulled the hair of the nurse at a health care facility.	No information provided.	Verdict set aside for other reasons and a new trial ordered.	No.

There are a few possible reasons why we found so few criminal cases despite the widespread accounts of violence against nurses in the workplace. First, the databases we searched, while the most comprehensive available, do not contain all legal judgments, including some from lower courts where access to court documents requires contacting individual court offices. One decision we found (*R v. Van Horlick* in 2020 which resulted in jail time for the offender) was not accessible on CanLII or Quicklaw. The research team requested the oral trial and sentencing reasons from the New Brunswick Provincial Court and were provided by the court (after fee payment) as untranscribed audio files. Further, not all criminal charges proceed to sentencing; for example, the accused may be offered diversion (where charges could be withdrawn or stayed in exchange for alternative actions like completing counselling or community service) or found unfit to stand trial. Thus, these 12 cases may not represent the full extent of criminal sentencing decisions in Canada where the victim of violence was a nurse but are nonetheless illustrative for what they reveal about how violence against nurses is treated within the criminal justice system.

These cases occurred before Canada's *Criminal Code* was amended to include the victim being a health care provider as a specific aggravating factor; however, even in the absence of statutory direction, courts have previously considered victimizing workers in other public-service, vulnerable, and high-risk jobs as an aggravating factor (e.g., a social worker in R v Dobbin, 2013 and a security guard in R v Banjoko, 2015). In three of the 12 cases, the judges made general comments about the victim being a nurse, while in two more recent cases, the judges considered the fact the victim was a nurse as an aggravating factor on sentencing. The fact that the victim was a nurse was not discussed in the remaining seven cases and thus not considered a factor at sentencing. More details about these cases and the criminal conduct involved can be found in Table 1.

In three of the 12 decisions, the judges made general comments about the victim being a nurse but stopped short of finding this was an aggravating factor. In one case, a judge in Newfoundland remarked that health care workers and patients should not be subject to workplace violence (R v Martin, 2017). Similarly, a judge in British Columbia said that the court would take note of the fact that workers who are providing public services, such as nurses, should not be attacked while on the job (R v Stard, 2016). In response to an assault on a police officer and a nurse, a judge in Alberta stated,

Police and health care workers are on the front lines. They serve society by performing critical functions. Yet, despite the respect that their services command of members of society, Mr. Gelenzoski chose to show the exact opposite. The message that the law will not tolerate the type of behaviour that Mr. Gelenzoski has demonstrated must be sent loudly and clearly and it must be sent not only for Mr. Gelenzoski, but for others who may think it is appropriate or acceptable to follow in his footsteps. (R v Gelenzoski, 2006, para 33)

In this case, while the victim's status as a nurse was not considered an aggravating factor, the judge emphasized the sentencing objective of denunciation, which generally leads to a longer, more significant sentence (Robitaille & Winocur, 2019).

In two decisions of the 12 cases we found (R v Mustafa, 2021 and R v Van Horlick, 2020), the judges considered the fact that the victim was a nurse when evaluating aggravating factors for sentencing. These were both recent cases in which the judges might have been aware of the House of Commons report and the proposed Criminal Code amendments, despite these not being enacted yet. In the reasoning provided in R v Mustafa (2021), the Ontario Court of Justice trial judge observed that the sexual assault occurred in the context of performing client care and treated it as an aggravating factor for sentencing. On appeal, the Superior Court of Justice upheld the sentence. The fact the victim was a nurse performing nursing services with a client was seen as an important aggravating factor and, according to the appeal judge, the “trial judge was correct” (para. 18) to consider this.

In R v Van Horlick (2020), the New Brunswick Provincial Court also considered the roles of the victims as nurses as an aggravating factor during sentencing for assault. The judge noted that nurses have the right to work in a safe environment and that a clear and unequivocal message needed to be sent to all members of society. The sentencing judge concluded that the aggravating factor of the victim being a nurse would mean the focus of sentencing would be on principles of denunciation and deterrence. During sentencing, the judge referred to a comment made by Van Horlick in pre-sentencing in which he had shown no remorse. Van Horlick had told the judge that he had not set out to hurt anyone but that he had been put in that position because the nurse manager had not done her job, and therefore she should be thankful that he had not killed her. In rejecting defence counsel's suggested conditional sentence, the judge concluded “I am not convinced that a conditional sentence would be a clear message to Mr. Van Horlick and to society that resorting to violence to solve problems is unacceptable and unlawful and that nurses and other employees in medical institutions have the right to work in a safe environment and be respected by their patients and members of their family” (R v Van Horlick, 2020, p. 0:31).^1^

### Occupational Health and Safety Law: Recent Amendments to the OHSA

The *Occupational Health and Safety Act* (OHSA) (1990) in the province of Ontario is a “public welfare statute that is intended to provide a minimum level of protection for the health and safety of workers” (Ontario [Ministry of Labour] v United Independent Contractors Ltd., 2011). Under the OHSA, employers are required to take every reasonable precaution to protect a worker. Ontario's highest provincial court, the Ontario Court of Appeal, has said that because the OHSA is protective legislation designed to promote public health, it should be interpreted generously (Ontario [Ministry of Labour] v Hamilton [City], 2002). Adjudicators have held that the extent of employer precautions taken must be assessed objectively (Toronto Police Assn. v Toronto Police Services Board, 2010).

In 2010, provisions were added to the OHSA to address workplace violence and harassment. These require, among other things, that employers assess the risks of workplace violence that may arise from the nature of the workplace, the type of work, or the conditions of work (s. 32.0.3), prepare and post “in a conspicuous place” a policy regarding workplace violence (s. 32.0.1(1)(b)), and reassess the risks of workplace violence as often as is necessary to protect workers (s. 32.0.5). Similar provisions were added around workplace harassment (s. 32.0.1). The rationale behind these amendments was that workplace violence is often foreshadowed and predictable and can be prevented if employers and supervisors seriously heed these signs of danger (Kingston [City] v. C.U.P.E., Local 109, 2011).

Further amendments to the OHSA were recently made as part of Bill 88 (2022), most of which came into effect on April 11, 2022. These amendments aimed to impose stricter penalties for non-compliance with the Act and compel greater organizational responsibility over the health and safety of workers. Specifically, the amendments increased the maximum fine from $100,000 to $1,500,000 (per offense) for directors or officers of corporations who fail to comply with the OHSA and to $500,000 for all other individuals (Bill 88, 2022). In addition to these substantially increased maximums, individuals found guilty of OHSA violations could face up to 12 months in jail (Bill 88, 2022). These amendments were necessary, in part, because of concerns that previous fines were an insufficient deterrent for employers and had been accepted as a “cost of business” (Ontario's Regulatory Registry, 2022).

Another amendment was to add a list of aggravating factors that must be considered when imposing penalties for OHSA violations; these factors include an offence that results in death, serious injury, or illness of one or more workers, an offence where the defendant commits the offence recklessly or shows a lack of remorse or has a record of prior non-compliance with the Act or its regulations, or an offence that was motivated by a desire to increase profits or reduce costs (Bill 88, 2022).

### OHSA Cases Involving Assaults Against Nurses

We reviewed OHSA cases that had occurred since the workplace violence provisions were added in 2010 and involved workplace violence against nurses. We found five cases where an employer was charged under OHSA that involved violence against nurses (see Table 2). Like our discussion of the criminal sentencing decisions, these five cases that proceeded to charges under the OHSA and prosecution of employers likely do not represent all OHSA cases involving workplace violence against nurses. The Ontario government can take enforcement action other than prosecution under the OHSA, including issuing a requirement or administrative order. Nonetheless, these cases are illustrative for what they reveal about how the occupational health and safety system treats violence in health care workplaces. We discuss these five cases according to three key circumstances that indicate severity of the workplace violence: first, some cases demonstrated patterns of violent behaviour; second, some cases involved multiple personnel; third, the same organizations were charged with multiple or consecutive violations. Finally, we discuss how the severity of penalties levied against employers varied despite these similar circumstances.

**Table 2.** OHSA Cases Involving Violence Against Nurses

Case name	Year	Facts	Charges	Decision	Commentary from judicial decision maker on victim being a nurse
The Brockville Mental Health Centre v The Ontario Ministry of Labour (See also Royal Ottawa Health Group – Brockville Mental Health Centre v Ontario Nurses Association, 2014, 2016)	2019	A psychiatric patient was admitted to BMCH and committed 15 assaults between August and October 2014. On the 15th assault, the patient repeatedly stabbed a nurse in the head and the neck (near the carotid artery) with a pen. The nurse suffered from PTSD and was unable to return to work. The patient was restrained but assaulted several other nurses by scratching, hitting, and biting. Another nurse was assaulted by a different patient in the same facility which resulted in a requirement for modified work duties. The hospital failed to complete a Critical Incident Report within 48 h as required by s. 51 of the OHSA. Additionally, BMCH did not conduct a full re-assessment of the risks of workplace violence.	The hospital was charged with contravening s. 32.0.3(4) of the OHSA by failing to assess the risks of violence, as often as is necessary to ensure that the workplace violence policy and the program for implementing the workplace violence policy continue to protect workers from workplace violence.	The hospital was fined $75,000 for the violation and imposed a 20% victim fine surcharge which was upheld on appeal.	The appellate judge held that public not-for-profit companies should not receive lesser fines; all workers deserve the same protections. The court found a connection between the failure to conduct a risk re-assessment and the series of assaults and concluded that the trial judge was entitled to consider it aggravating that the hospital's failure contributed to the series of assaults.
Ministry of Labour v Royal Ottawa Health Care Group	2016	A psychiatric patient assaulted six health care personnel, three RPNs, one RN, and two PCAs in a single altercation. The staff struggled to call for assistance by calling for a “code white” and experienced bodily harm while trying to escape and subdue the assailant. The offender had assaulted health care workers at the facility two other times but had little to no subsequent restrictions on their movements around the facility.	1. Failing as an employer to develop and maintain a program to implement the workplace violence policy that contains measures and procedures for summoning immediate assistance when workplace violence occurs contrary to s. 32.0.2(2)(b) 2. Failing to provide information, instruction, and supervision to a worker to protect the health or safety of the worker, contrary to s. 25(2)(a). 3. Failing as an employer to take every precaution reasonable in the circumstances for the protection of the worker contrary to s. 25(2)(h)	The hospital was acquitted of all charges.	The Justice of the Peace was critical of the nurses and suggested that the reason the offender was able to assault the health care workers was because they fled. The Justice of the Peace stated: “The question in the Court's mind is: Where are the other two individuals? [One RN] in the nursing station calls Code White and *goes into hiding*. [The second RN] hides behind the door of the nursing station and *allows* [the offender] to come in to get at [the RPN].” [emphasis added].
Ministry of Labour v The Centre for Addiction and Mental Health (CAMH)	2016	A psychiatric patient assaulted a nurse from behind, pushing her to the floor and kicking her repeatedly in the head. Two other nurses on the unit responded to the incident, one aided the first victim in running to safety while the second called for assistance. The primary and secondary victims suffered from significant physical and psychological injury as a result of the assault and were both unable to return to work.	CAMH pled guilty to contravening section 25(1)(c) of the OHSA by failing as an employer to ensure that the measures and procedures prescribed by Section 8 of the Health Care and Residential Facilities Regulation were carried out in the workplace. Section 8 requires that hospitals must “establish and put into effect measures and procedures for the health and safety of workers.”	The CAMH was fined $80,000 for the violation. Aggravating factors included: - CAMH contravened the OHSA twice in 2009. - There was potential for the OHSA violation to lead to harm Mitigating factors included: - The hospital pleaded guilty, which saved public expense and the injured nurses were not required to testify. - CAMH was a public institution fulfilling a public responsibility.	None
Ontario Nurses’ Association v Southlake Regional Health Centre (See also Ontario Newsroom, 2020)	2019	A psychiatric patient assaulted and injured two hospital workers, a nurse, and a security guard.	SRHC faced nine charges with a penalty of $1.5 million for each charge. Seven charges related to taking reasonable precautions to protect workers. Two charges related to the provision of information and supervision to workers.	SRHC pled guilty to two charges and the remaining seven were dismissed. SRHC was fined $80,000 (Ontario Newsroom, 2020) and a victim surcharge of 25% which equated to a total penalty of $100,000.	None
Ontario Nurses’ Association v Southlake Regional Health Centre	2021	Three further incidents occurred in 2020.	SRHC faced five charges with a penalty of $1.5 million for each charge. The CEO faced one charge for failing to act to ensure the safety of workers after having prior knowledge of a patient's violent behaviour. This charge included fines of up to $100,000 and up to 12 months imprisonment.	The charges against SRHC and the charge against the CEO were dismissed.	None

In two cases, violent patient behaviour occurred repeatedly over time. For example, in Brockville Mental Health Centre v the Ontario Ministry of Labour (2019), a patient assaulted staff on 15 separate occasions over the course of a few months. Ultimately, the patient stabbed a nurse repeatedly in the head and neck with a pen. As a result, this nurse suffered from post-traumatic stress disorder (PTSD) and could not return to work. In Ministry of Labour v Royal Ottawa Health Care Group (2016), a patient was admitted to The Royal Ottawa Mental Health Centre recovery unit from another ward with a history of two previous assaults. Similarly, at the Centre for Addiction and Mental Health, a nurse was severely assaulted by a patient with a known history of violence (Ministry of Labour v The Centre for Addiction and Mental Health, 2016).

Three cases also demonstrated involvement of multiple health care personnel. At the Royal Ottawa Mental Health Centre, the patient in question entered the room reserved for health care personnel and put their hands on a nurse and asked to embrace them. A second nurse attempted to get the patient to leave the nursing area but was charged by the patient and slammed against the glass door, which then broke. A third nurse called a “Code White” overhead and then fled, along with a fourth nurse, to the nursing station. The patient entered the nursing station before the nurses could close the door and strangled and hit the fourth nurse before throwing her to the ground. While this was occurring, one of the other nurses made a second, but unsuccessful, attempt to call a Code White before running back to assist the nurse being assaulted. That nurse then became the victim of further assault as the patient grabbed their head and hit it against the door. At this point, additional health care staff arrived and were able to temporarily subdue the patient, who then struck one of the staff members in the head and attempted to bite another once breaking free. One of the nurses called 911 and eventually the patient was subdued by two staff members and another patient on the unit. Only after the patient was restrained did the Code White respondents arrive. At the Centre for Addiction and Mental Health, a patient assaulted a nurse from behind, pushing her to the floor and kicking her repeatedly in the head. That nurse could not access her personal alarm device but two other nurses could hear and respond to the assault. One of these nurses left to call for help while the other tried to intervene to help the victim run toward safety. Southlake Regional Health Centre faced charges after two hospital workers, a registered nurse and a security guard were injured after a violent incident involving a patient on January 17, 2019 (Kelly, 2020).

Some of these cases involved multiple or consecutive violations by the same employer after initial reports of workplace violence were met with insufficient responses from the employer. In the case involving Brockville Mental Health Centre, the hospital responded to repeated incidents with some additional security measures but did not conduct a complete re-assessment of the risks. The hospital failed to submit the required Critical Incident Report within 48 h (Royal Ottawa Health Group – Brockville Mental Health Centre v ONA, 2014) and conducted a full reassessment of the risks of workplace violence only once when ordered to by an inspector from the Ministry of Labour (The Brockville Mental Health Centre v. The Ontario Ministry of Labour, 2019). The Centre was subsequently charged and convicted for failing to assess the risks of violence as often as necessary to ensure that the workplace violence policy protected workers. The Centre was ordered to increase security presence, provide additional staff training, update policies, and establish regular communication with staff about the level of risk of violence in the unit (Royal Ottawa Health Care Group – Brockville Mental Health Centre v ONA, 2016). The Centre was fined $75,000 for the violation and a 20% victim surcharge was imposed, which was upheld in an appeal, resulting in a total fine of less than $100,000. The fact that the victim was a nurse was not given particular consideration; however, the judge considered the Centre's failure to conduct a risk reassessment an aggravating factor that contributed to the series of assaults (The Brockville Mental Health Centre v. The Ontario Ministry of Labour, 2019).

The Royal Ottawa Mental Health Centre faced three charges relating to maintaining policies and procedures for summoning assistance, failing to provide information, instruction, and supervision to a worker, and failing to take every reasonable precaution for the protection of its workers. In this case, The Royal Ottawa Mental Health Centre was acquitted of all charges (Ministry of Labour v Royal Ottawa Health Care Group, 2016). The Justice of the Peace suggested that the reason the patient could assault the nurses was due to their choice to flee to safety rather than assist their colleagues, not because the hospital did not have adequate measures in place to protect the health care workers. In reference to this incident and the charges, the Justice of the Peace focused on the responsibility of the nurses by stating,

The question in the Court's mind is: Where are the other two individuals? [The third nurse] in the nursing station calls Code White and goes into hiding. [The fourth nurse] hides behind the door of the nursing station and allows Patient X to come in to get at [the second nurse]. What would have happened had she closed the door after [the first two nurses] had entered into the station? Could it have been prevented? We also hear the evidence of [the third nurse], ‘If she had closed the door, he would not have been able to get into the nursing station.’ Six employees are available to respond within a short period of time of the incident starting and only four truly respond. (Ministry of Labour v Royal Ottawa Health Care Group, 2016, p. 73, para 271-272)

In the Ministry of Labour v The Centre for Addiction and Mental Health (2016) case, involving an assault on a nurse, the Centre pleaded guilty to failing to “establish and put into effect measures and procedures for the health and safety of workers” and was fined $80,000 (Ministry of Labour v The Centre for Addiction and Mental Health, 2016). Southlake Regional Health Centre (Ontario Nurses’ Association v Southlake Regional Health Centre, 2019; 2021) faced seven charges in relation to the requirement to take every reasonable precaution to protect workers, while two of the alleged violations were regarding the provision of required information and supervision to workers (Kelly, 2020). Southlake pleaded guilty to two charges and the remaining seven were dropped (Weisz, 2022). The guilty plea was in relation to charges regarding the ability of staff to communicate with their unit members and points of entry and exit on the unit (Ontario Newsroom, 2020). As a result of the guilty plea, Southlake Regional Health Centre was fined $80,000 (Ontario Newsroom, 2020) and a 25% victim fine surcharge, which equated to a total penalty of $100,000 (Weisz, 2022).

In response to the serious nature of the incident that occurred in 2019, the then-CEO of Southlake Regional Health Centre released a statement on the Centre's website stating, “The safety of everyone who works and receives care at Southlake is a top priority. We want everyone to feel safe at the hospital… Thankfully violent incidents are incredibly rare” (Krystal, 2020, p. e1). Within the months that followed, however, three further incidents took place that resulted in five additional charges against Southlake Regional Health Centre and one against its former CEO, each with a penalty of up to $1.5 million (Weisz, 2022). The CEO had participated in “shadow shifts” during which she observed a patient being aggressive who, in the days following, assaulted a nurse. The CEO responded to the event in an email to the ONA stating she “wasn’t surprised by the incident as she had prior knowledge of the patient in question” (Weisz, 2022, p. e1). Despite their prior knowledge of the risk and previous charges, the Centre and the CEO were acquitted of all charges.

## Discussion

In the CFNU's study in 2019, almost half of nurses in Canada reported exposure to physical assault 11 or more times over their careers (Stelnicki et al., 2020, p. 47). Given the reports of escalating violence to which health care workers have been exposed since the COVID-19 pandemic began (Government of Canada, 2021), the number of nurses who have experienced or witnessed physical assault is likely to have risen since these results were released. Despite these statistics demonstrating rampant violence against nurses, we could not find many cases that resulted in criminal charges and sentencing decisions. Even amongst those cases tried in the court of law, the victim's status as a nurse was not consistently considered an aggravating factor in sentencing, lending support to the decision by the Canadian government to amend the Criminal Code to require this consideration at sentencing.

One potential reason the vulnerability of nurses has not translated into an aggravating factor earlier is due to a lack of recognition of the inherent danger of the nursing profession. For example, in a 2015 decision, a labour arbitrator wrote that “unlike police work, health care work is not an inherently risky profession. While there is some risk, people do not become health care providers with the knowledge and expectation that they are entering a dangerous profession” (Prairie North Health Region and CUPE, Local 5111 (Employee Name Tags), Re, 2015). However, nurses are three times more likely to experience violence than any other professional group (Registered Nurses Association of Ontario, 2008).

One reason we were unable to find more cases of violence against nurses may be that acutely ill patients may not typically be held accountable for their violence against nurses and other caregivers, and thus not prosecuted. Passive acceptance of violence against nurses persists in the legal system, perhaps because “patients are [considered] ill and [therefore] cannot control themselves” (R v Gelenzoski, 2006). This mentality provides a rationale for the lack of court cases and has perhaps led to an expectation that nurses are responsible for managing the risk of physical violence in the workplace, considering it “part of the job” (Henry, 2022). This is evidenced in the case of the *Ministry of Labour v Royal Ottawa Health Care Group* (2016) where all charges against the health centre were dropped and the nurses were criticized for seeking personal safety during a violent assault.

The passing of Bill C-3 in 2021 amended the Criminal Code, making it an offence for a person to “engage in any conduct with the intent to provoke a state of fear” in health professionals performing their duties. The Bill also increased the maximum sentence for intimidation or assault against health care workers from five to ten years and added aggravating factors that may be used during sentencing of such crimes (Bill C-3, 2021). It could be argued that the new legislation is inadequate to address workplace violence. Specifically, the amendment does not add protections for health care workers, given that it was already a crime to block access to a hospital, or intimidate, harass, threaten, or assault health care professionals. In the time that has passed since Bill C-3 became law, there have been many reports of intimidation and violence against health care workers amidst anti-government protests such as the “Freedom Convoy” (Duong & Vogel, 2022; Kaufmann, 2022; Payne, 2022) yet these amendments to the Act have not been widely enforced and do not appear to make an impact on the protection of nurses across Canada. On the contrary, during the anti-vaccination protests, health care workers were encouraged to avoid wearing their work uniforms so they would be less identifiable to possible assailants (Duong & Vogel, 2022; Katawazi, 2022). Once again, responsibility was placed on nurses and other health care professionals to protect themselves rather than directly addressing unsafe work environments.

Bill 88 (2022) amended the OHSA to increase maximum fines to the corporation and the directors to $1.5 million per charge instead of the previous $100,000 maximum. Despite the increased sanctions available within the law, in practice the courts remain reluctant to impose severe consequences on the employer for failing to protect their staff. For instance, under the amendments made to the OHSA, Southlake Regional Health Centre faced a combined total of 14 charges with a potential fine of up to $21 million and the CEO faced a potential fine of $1.5 million and 12-month imprisonment for failing to protect employees. The health centre pleaded guilty to two charges and the rest were dropped, resulting in a total penalty of $100,000 (Ontario Newsroom, 2020). This penalty was the most significant sentencing of all the employers tried for violations under the OHSA (1990) included in this study. Despite the intended impact of Bill 88, the results of the 2019 case against Southlake Regional Health Centre revealed that the penalties had failed to motivate the centre to improve protections for their employees, despite ongoing assaults against nurses at the centre. A penalty of many millions of dollars could be viewed as significant incentive to invest in improved workplace safety provisions, whereas a fine of $100,000 could be more easily absorbed by the organization. The outcomes of the cases tried for violations under the OHSA (1990) in relation to violence against nurses, including those that based penalties upon the then-impending Bill 88 (2022), showed little indication of employers being held to account for the safety and protection of their workers. Instead, the outcomes, which at times included criticism of staff reactions under dangerous and terrifying circumstances, tended to be highly sympathetic to the employer; this may dissuade victims of violence from coming forward to give evidence against their employer, further perpetuating a culture that normalizes violence against nurses as “part of the job.”

The court documents we examined did not outline what actions the employers should have taken to avoid the risk but considered whether the employers were compliant with legislation that ensures employee safety. The fact that two of these employers had successive cases before the courts raises questions in this regard, and the increased penalties in the amended legislation indicate that, at least in the mind of government, employers needed the threat of more severe economic sanctions to invest in improving staff safety.

## Conclusion

Violence against nurses is a persistent and widespread issue globally, with incidences of violence increasing in frequency since the pandemic. In this article, we reviewed how the Canadian legal system has characterized and addressed this phenomenon and provided an analysis of Canadian court cases involving violence against nurses. We included both criminal cases, where a perpetrator is charged by Crown Counsel on behalf of society, and cases under workplace law, where either the Ministry of Labour or a union is bringing the case against the employer. In both categories of cases, the incidents that are reported are egregious with significant injury and trauma to the nurse(s) and other staff. Under criminal law, the limited number of cases we could find with oral or written sentencing decisions show that, historically, the fact that a victim was a nurse was not always considered an aggravating factor on sentencing. Now that this is a specific aggravating factor under Canada's *Criminal Cod*e, it will be interesting to track the weight that judges give to this factor when exercising their judicial discretion in applying an appropriate sentence. With respect to employment law, it appears that despite the government's efforts to substantially increase the deterrence factor under legislation with significantly increased fines for employers who fail to protect their employees from injury, courts remain reluctant to impose such sanctions. In these cases, it will also be important to track the impact of harsher penalties over the longer term.

Meanwhile, the new federal legislation covering violence against health care workers has not significantly expanded the previous provisions under the Criminal Code; nor was the new provision consistently invoked when charging those who intimidated health workers during the widespread COVID-19 protests in winter 2022. It also remains to be seen if there is any willingness to continue to charge offenders under this Act. For those assailants who are prosecuted and found criminally responsible, and for those employers who do not meet their expectations for workplace safety, ongoing legal efforts to combat the widespread acceptance of workplace violence in health care, and specifically against nurses, continue to be acutely needed.

This paper reports on an analysis of legal cases involving violence against nurses. The court system is complex; cases are challenging to find and there are variations in employment law among jurisdictions. This paper offers practitioners, researchers, and policy makers an overview of the court response to individuals and organizations that seek redress for violence that befalls nurses in the course of their work. We hope that awareness of these types of legal decisions will aid policy makers and nurse leaders to hold the courts accountable with respect to sentencing and put pressure on employers to ensure they are fully compliant with existing legislative requirements to provide a safe working environment for all their staff, including nurses.

## Notes

1. The nurse manager sued Van Horlick in civil court, seeking more than $1.5M in compensation for lost income and future earnings, and for damages. On March 2, 2022, Van Horlick was ordered to pay $1,357,847 including costs. Van Horlick did not file a statement of defence and failed to attend court (Magee, 2022).
